# A New Subcarrier Allocation Strategy for MIMO-OFDMA Multicellular Networks Based on Cooperative Interference Mitigation

**DOI:** 10.1155/2014/652968

**Published:** 2014-02-10

**Authors:** Panagiotis K. Gkonis, Maria A. Seimeni, Nikolaos P. Asimakis, Dimitra I. Kaklamani, Iakovos S. Venieris

**Affiliations:** School of Electrical and Computer Engineering, National Technical University of Athens, Heroon Polytechniou 9, Zografou, 15773 Athens, Greece

## Abstract

The goal of the study presented in this paper is to investigate the performance of a new subcarrier allocation strategy for Orthogonal Frequency Division Multiple Access (OFDMA) multicellular networks which employ Multiple Input Multiple Output (MIMO) architecture. For this reason, a hybrid system-link level simulator has been developed executing independent Monte Carlo (MC) simulations in parallel. Up to two tiers of cells around the central cell are taken into consideration and increased loading per cell. The derived results indicate that this strategy can provide up to 12% capacity gain for 16-QAM modulation and two tiers of cells around the central cell in a symmetric 2 × 2 MIMO configuration. This gain is derived when comparing the proposed strategy to the traditional approach of allocating subcarriers that maximize only the desired user's signal.

## 1. Introduction

Recent advantages in wireless communications include among others the adoption of the Orthogonal Frequency Division Multiple Access (OFDMA) physical layer protocol for the Long Term Evolution (LTE) of the currently established third generation (3G) wireless networks [1]. In OFDMA, the available spectrum is divided into a number of narrowband orthogonal subcarriers that can be dynamically allocated to mobile stations (MSs), thus providing bandwidth on demand and increased data rates. The use of orthogonal subcarriers allows their spectrum to overlap; hence, the overall spectrum efficiency is increased. Moreover, the insertion of a cyclic prefix (CP) at the beginning of the OFDM symbol can significantly reduce intersymbol interference (ISI) in cases of multipath propagation.

Another challenging research field in the area of next generation broadband wireless networks is the design and implementation of efficient transceiver architectures that further increase the spectrum efficiency of the network without additional spectrum requirements. The integration of Multiple Input Multiple Output (MIMO) transmission technology in OFDMA multicellular networks is a promising research field, as it tries to combine the individual advantages of these two techniques and identify practical limitations [2].

The goal is to find the appropriate subcarrier allocation algorithm, transceiver architecture, and transmission power per MS that improve network performance and at the same time minimize the overall signaling burden. However, this three-dimensional optimization problem cannot be solved analytically, especially in the case of highly loaded networks. Therefore, suboptimal optimization algorithms have been studied thoroughly in the literature, wherein the vast majority optimization is employed over decomposed problem formulations (e.g., separate subcarrier/power allocation).

In this context, in [3] an overview of Space Time (ST), Space Frequency (SF), and Space Time Frequency (STF) coding for fourth generation (4G) MIMO-OFDM broadband wireless systems is provided. Performance results show that STF coding can achieve the maximum diversity gain in an end-to-end MIMO-OFDM system over broadband wireless channels. In [4], a rate dependent STF code is proposed and evaluated for a two-user case scenario. The derived results indicate that joint code design significantly outerperforms single-user code design for high values of signal to noise ratio (SNR). Other studies have focused on resource allocation for OFDMA networks: in [5], the resource allocation problem is effectively approached with a chunk-based resource allocation scheme, that is, optimal chunk, power, and bit allocation. A digitization process is proposed by considering the digital nature of bits/symbol/subcarrier, which leads to better average throughput of this dynamic power allocation compared to the fixed power allocation scheme. In [6], a low-complexity suboptimal algorithm that separates subchannel and power allocation is presented and evaluated. In the proposed algorithm, subchannel allocation is first performed by assuming an equal power distribution. An optimal power allocation algorithm then maximizes the sum capacity while maintaining proportional fairness. In [7], three adaptive slot allocation algorithms for wireless OFDMA systems have been introduced. The proposed algorithms have improved performance with respect to a static allocation scheme that does not take into account the different channel conditions of the users.

In the same context (i.e., resource allocation), various approaches have been proposed that are employed in cases of MIMO orientations as well. In [8], a two-step resource allocation scheme is presented, where the system is statically divided into a number of disjoint clusters. This scheme results in intercluster interference mitigation and maximization of the sum utility function of all MSs in the cluster, under per-sector power constraints. In the same context, in [9] an optimal resource allocation algorithm is developed on the basis of the standard graph theory and the Lagrangian relaxation. Moreover, a lower-complexity suboptimal algorithm is introduced as well. In [10], a resource allocation algorithm for MIMO multicast systems over frequency-selective channels has been introduced, that provides significant gain over total throughput. In [11], a genetic algorithm for solving rate adaptive resource allocation problem with proportional rate constraints for MIMO-OFDMA systems was presented and evaluated.

In all the above studies, however, limited network orientations were taken into account (i.e., reduced number of active sectors and limited number of subcarriers or active users per sector). Moreover, power and subcarrier allocation were treated as two separate problems. In this study, the goal is to evaluate the performance of a new subcarrier allocation strategy for MIMO-OFDMA networks that maximizes the desired MS's signal and at the same time minimizes the interference caused to the rest of the network. This is performed through cooperative interference mitigation among adjacent Base Stations (BSs). In this context, a hybrid system-link level simulator has been developed, where both MIMO transmission in the physical layer and subcarrier allocation in the Medium Access Control layer are taken into consideration. Up to two tiers of cells around the central cell are also considered and increased loading per cell.

The rest of this paper is organized as follows: in Section 2 the multicellular MIMO-OFDMA simulator is described, while in Section 3 the MIMO-OFDMA system model along with the proposed subcarrier allocation algorithm is presented. The derived results are discussed in Section 4, where the performance of the proposed subcarrier allocation strategy is highlighted, while concluding remarks are made in Section 5.

## 2. Multicellular MIMO-OFDMA Simulator

**Figure 1:**
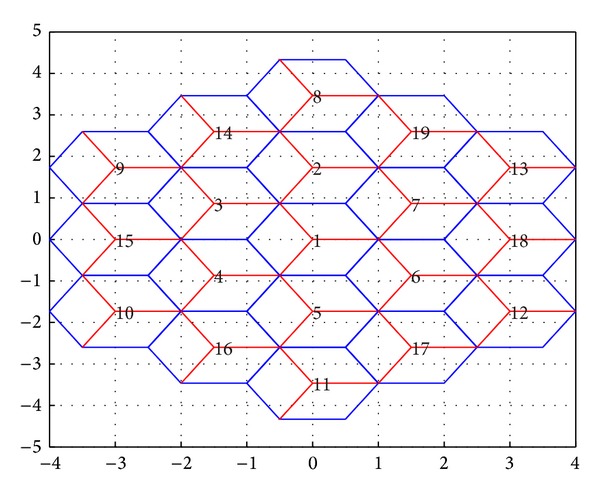
Cellular configuration (axes in kms).

A MIMO-OFDMA system with up to two tiers of cells around the central cell (Figure 1) and three sectors per cell is considered. All sectors employ conventional 120° sectors with radiation patterns as specified in [12]:

(1) $$\begin{array}{c} f\left(\varphi \right)=G_{b}-\min\left[12{\left(\frac{\varphi -\varphi_{s}}{\varphi_{3\;\text{dB}}}\right)}^{2},A_{m}\right], \end{array}$$

for *φ*_*s*_ − 60° ≤ *φ* ≤ *φ*_*s*_ + 60°. In (1), *φ*_*s*_ ∈ {60°, 180°, 300°} is the pointing direction of the specific sector, the antenna gain *G*_*b*_ equals 14 dBi, the 3 dB beamwidth of the antenna pattern (*φ*_3 dB_) is 70°, and the front-to-back ratio (*A*_*m*_) is 20 dB. The macrocell path loss is based on the Okumura-Hata model with a BS height of 30 m, an MS height of 1.5 m, and a carrier frequency of 2 GHz [13]:

(2) $$\begin{array}{c} \text{PL}=137.4+35.2\;\text{lo}{\text{g}}_{10}\left(d\right), \end{array}$$

where PL is the path loss in dB and *d* is the distance in kms. The employed simulator is semistatic; hence, MSs' locations do not change during a simulation run. MSs enter the network sequentially, following a uniform distribution. An MS is connected to the BS with the lowest path loss (including shadowing and antenna radiation patterns). Once a new MS is accepted, a number of subcarriers are allocated for downlink transmission, based on the proposed subcarrier allocation algorithm described in Section 3. This procedure is repeated for as long as the blocking probability (defined as the ratio of rejected MSs to the total number of MSs that tried to access the network) remains below a predefined threshold. In all simulations, a total number of 128 available subcarriers per cell's sector are assumed for downlink transmission in a bandwidth of 10 MHz and a central frequency of 2.5 GHz.

Once the effective MSs as well as the relevant parameters (i.e., positions, total losses, etc.) are defined, link level simulations take place. Denoting by *D* the total number of drops for each network orientation and by *F* the number of independent channel realizations per MS, then for the sake of simplicity it is assumed that *D* = *F*. This practically means that each Monte Carlo (MC) simulation consists of a specific MSs' distribution and one channel realization per MS. Typically, more than one thousand MC snapshots per network orientation may be required for converging simulation scenarios.

## 3. MIMO-OFDMA System Model and Problem Formulation

Assuming an arbitrary MIMO configuration with *M*_*t*_ transmit antennas and *M*_*r*_ receive antennas, then the transmitted *M*_*t*_ *x*1 signal for the *n*th MS is expressed as a sum of individual products:

(3) $$\begin{array}{c} x_{n}\left(t\right)=\sum_{s\in S_{n}}\sqrt{p_{n,s}}w_{n,s}X_{n,s}e^{j2\pi f_{s}t},\;0<t<T, \end{array}$$

where *j* is the imaginary unit, *p*_*n*,*s*_ is the power of the *n*th MS allocated to the *s*th subcarrier, *T* is the duration of the OFDM symbol, and **x**_*n*_(*t*) and *X*_*n*,*s*_ are the transmitted vector signal in time domain and the *s*th symbol of the *n*th MS drawn from a predefined constellation, respectively (i.e., QPSK, QAM, etc.). It is assumed that downlink transmission for the *n*th MS takes place using the subcarriers in set *S*_*n*_, with *f*_*s*_ being the corresponding frequency for the *s*th subcarrier.

Considering diversity combining transmission mode, then *X*_*n*,*s*_ is replicated in *M*_*t*_ antennas properly weighted through the factor **w**_*n*,*s*_. The signal in (3) is sampled at *S* discrete time intervals, where *S* is the total number of available subcarriers. Therefore, the actual transmitted signal for a specific symbol period is given by [14]

(4) $$\begin{array}{c} x_{n,l}\left[m\right]=\sum_{s\in S_{n}}\sqrt{p_{n,s}}w_{n,s}X_{n,s,l}e^{j2\pi ms/S},\;0<m<S-1, \end{array}$$

where *X*_*n*,*s*,*l*_ denotes the corresponding symbol (i.e., *l*, where 0 < *l* < *∞*) transmitted from the *s*th subcarrier.

During transmission, the signal undergoes the effects of shadowing, fast fading, and Multiple Access Interference (MAI) from the other MSs in the cellular orientation allocated that have been allocated with the same set of subcarriers. Hence, the received *M*_*r*_ *x*1 signal for each subcarrier after matched filtering and Discrete Fourier Transform (DFT) operation can be expressed as (for simplicity only the first symbol period has been considered)

**Figure 2:**
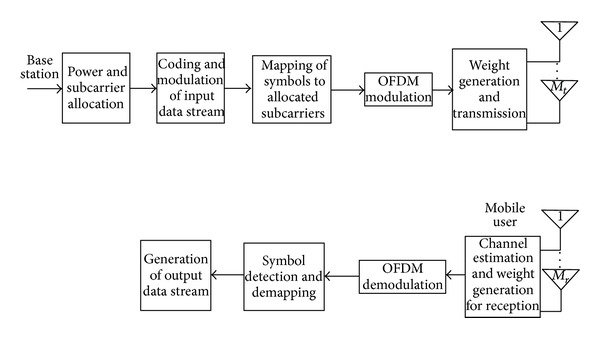
MIMO-OFDMA transceiver general block diagram.

(5) Yn,s=(pn,sTLn,sec(n))Hn,sec(n),swn,sXn,s +∑n′≠n,s∈Sn′N(pn′,sTLn,sec⁡(n′))Hn,sec⁡(n′),swn′,sXn′,s +noisen,s,

where TL_*n*,sec(*n*)_ are the total losses of the *n*th MS relevant to its serving effective sector including shadowing and antenna radiation patterns and **H**_*n*,sec(*n*),*s*_ is the *M*_*r*_ *xM*_*t*_ channel matrix (flat Rayleigh fading) for the *s*th subcarrier relevant to sec (*n*) while **n** **o** **i** **s** **e**_*n*,*s*_ is the corresponding *M*_*r*_ *x*1 additive white Gaussian noise. In (5), the middle term denotes MAI where only the interfering MSs (indexed as *n*′) that have been allocated with the *s*th subcarrier are considered. In order to coherently combine the individual signals received from the *M*_*r*_ antennas, Maximal Ratio Combining (MRC) is performed. Hence, the signal in (5) is multiplied by **r**_*n*,*s*_ = (**H**_*n*,sec(*n*),*s*_ **w**_*n*,*s*_)^*H*^ [13]. The output signal is then demodulated based on a given signal constellation. The overall transceiver architecture is depicted in Figure 2.

The Signal to Interference plus Noise Ratio (SINR) for the *n*th MS and *s*th subcarrier for a specific channel realization can be easily derived from (5) assuming independent transmitted streams among MSs:

(6) SINRn,s=(pn,swn,sHHn,sec(n),sHrn,sHrn,sHn,sec(n),swn,sTLn,sec(n)) ×(∑n′≠n,s∈Sn′pn′,swn′,sHHn,sec(n′),sHrn,sHrn,sHn,sec(n′),swn′,sTLn,sec(n′)   +rn,sHrn,sIo)−1.

In (6), *I*_*o*_ is the thermal noise power. Therefore, the optimal solution to resource allocation problem assuming a fixed data rate per MS would be to find the appropriate transmission vectors (i.e., **w**_*n*,*s*_), downlink transmission power per MS and subcarrier (i.e., *p*_*n*,*s*_), and allocated set of subcarriers per MS that maximize SINR_*n*,*s*_ over all MSs and subcarriers, subject to the following constraints:

(7) $$\begin{array}{c} {||w_{n,s}||}_{F}=1,\\ 0<\sum_{s\in S_{n}}p_{n,s}<p_{\max},\\ \sum_{n=1,n\in k}^{N}U_{n}\leq S,\;\text{for}\;\;1\leq k\leq K, \end{array}$$

where ||**x**||_*F*_ denotes the Frobenius norm of vector **x**. In the second condition of (7), the downlink transmission power per MS over all allocated subcarriers should not exceed its maximum allowed value. In the third condition, denoting by *U*_*n*_ the number of subcarriers allocated to the *n*th MS, the total number of subcarriers per effective sector (denoted by *k*) should remain below *S* in order to avoid link outage.

Since the above problem cannot be solved analytically due to the complexity of the cellular orientation (i.e., increased number of cells, MSs per cell, subcarriers per MS, etc.), an alternate approach is proposed that maximizes the desired MS's signal and at the same time minimizes the total amount of interference caused to the network. Considering two MSs (i.e., *n* and *n*′), the interference that the *n*th MS causes to the *n*′th MS (also referred to as jamming) will be given by

(8) $$\begin{array}{c} J_{n^{\prime },n,s}=p_{n,s}w_{n,s}^{H}\frac{H_{n^{\prime },\text{sec}\left(n\right),s}^{H}H_{n^{\prime },\text{sec}(n),s}}{\text{T}{\text{L}}_{n^{\prime },\text{sec}(n)}}w_{n,s}. \end{array}$$

Note that in (8) the matrix **H**_*n*′,sec(*n*),*s*_ denotes the channel to the *n*′th MS from the *n*th MS's serving sector. For the sake of simplicity, it is assumed that ||**r**_*n*′,*s*_||_*F*_ = 1 for all MSs and subcarriers.

From (8), it follows that the desired MS's signal power to the total amount of jamming and noise will be given by:

(9) SJNRn,s =(wn,sH(Hn,sec⁡(n),sHHn,sec⁡(n),s)wn,sH)  ×(wn,sH(∑n′≠n,s∈Sn′Hn′,sec⁡(n),sHHn′,sec⁡(n),s       ×(TLn,sec⁡(n)TLn′,sec⁡(n))+IoTLn,sec⁡(n))wn,s)−1.

**Figure 3:**
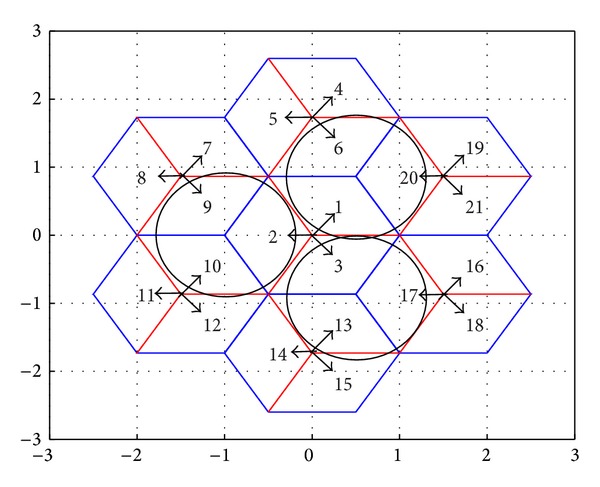
Cellular configuration and sets of adjacent sectors.

In (9), SJNR_*n*,*s*_ denotes the Signal to Jamming plus Noise Ratio for the *n*th MS and *s*th subcarrier which is independent of *p*_*n*,*s*_. Having defined the SJNR ratio, our proposed subcarrier allocation strategy is described in Algorithm 1. Note that **C**_BS_ denotes either seven or nineteen sets (i.e., for one and two tiers of cells around the central cell, resp.) that correspond to the available subcarriers per BS, **C**_sec_ denotes the available subcarriers per effective sector, and **C** **A** **S** denotes the joint available subcarrier set of a sector and its adjacent sectors. With respect to Figure 3, where for simplicity only the first tier of cells around the central cell is presented, for each sector in the network the set of its adjacent sectors is defined based on the pointing directions of the radiation patterns: for the first sector of the first BS, for example, its adjacent sectors will be the third sector of the second BS (i.e., sector 6 in Figure 3) and the second sector of the seventh BS (i.e., sector 20 in Figure 3). For this subset of sectors intracarrier interference is expected to be increased due to the pointing directions of the corresponding antenna elements [12].

Once all relevant parameters are initialized, for each MS that tries to access the network, the available set of subcarriers is first defined, based on the subcarrier availability of its sector and its adjacent sectors (i.e., joint set **C**_sec,*k*_∩**C** **A** **S**_*k*_ in Step 2, where **C**_sec,*k*_ denotes the available set of subcarriers in the *k*th sector). In order to minimize intercell interference, for each allocated subcarrier in a sector a virtual allocation takes place in its adjacent sectors as well: **C** **A** **S**_AS(*k*)_ ← **C** **A** **S**_AS(*k*)_∖SC, where AS denotes the set of adjacent sectors (with respect to the previous case AS(1) = {6,20}). Therefore, the first MSs, for example, in the first sector and its adjacent sectors will have different sets of allocated subcarriers and hence zero intercell interference. In this case, subcarrier allocation is based on the maximization of the corresponding channel matrix (i.e., line 6 of Algorithm 1). For the upcoming MSs, the subcarrier allocation is based either on the set of available subcarriers of their serving BS or on the available subcarriers of their serving sector (Step 3, lines 14-15 of Algorithm 1). The first case ensures reduced intercell interference, since among the three sectors of a specific BS there is 20 dB attenuation [12]. In both cases, subcarrier allocation is performed now according to SJNR maximization (i.e., line 19 of Step 4).

Afterwards, the transmit weight vectors are calculated, based on the maximum eigenvalue of the channel matrix. Namely, **X**(*λ*_*m*_(**A**)) is the eigenvector of matrix **A** corresponding to its maximum eigenvalue, denoted by *λ*_*m*_(**A**). Power control is also performed in Step 5. Throughout this procedure, the following notation has been used: **p**_*s*_ denotes the |*N*_*s*_ | *x*1 vector of downlink transmission powers for the *s*th subcarrier (i.e., |*X*| denotes the number of elements in set *X*) and **R**_*s*_ is the set of candidate MSs to be removed while MS_*r* is the MS that is removed from the network. Moreover, **1**() returns a vector matrix of ones. Finally, SINR_*s*_ denotes the SINR value for the *s*th subcarrier for acceptable Quality of Service (QoS). Note that power control procedure is performed for the subcarriers that have been allocated to the *n*th MS. In this case, matrices **A**_*s*_ and **B**_*s*_ can be easily formulated from (6), assuming common SINR requirement for all MSs of the *s*th subcarrier (i.e., SINR_*s*_). Therefore, a linear |*N*_*s*_ | ×|*N*_*s*_| system can be formulated and solved for the downlink transmission powers of the |*N*_*s*_| MSs of the *s*th subcarrier. If, however, the solution of this system does not result in realistic values for the elements of **p**_*s*_ (i.e., either negative values or values greater than *p*_max⁡_), then MSs are removed from the network until the second condition of (7) is satisfied for all MSs that have been allocated with the *s*th subcarrier.

## 4. Results and Discussion

### 4.1. Numerical Results

In order to evaluate the performance of the proposed subcarrier allocation strategy, MC simulations were performed in a network topology with up to two tiers of cells around the central cell. All simulation parameters are summarized in Table 1. In all simulation scenarios, QPSK and 16-QAM modulation types per subcarrier have been considered; hence, for 78.125 KHz subcarrier spacing a requested bit rate of 156.25/312.5 Kbps per subcarrier is assumed. MSs enter the network sequentially, as long as the ratio of rejected MSs to the total number of MSs that tried to access the network remains below a predefined threshold. In all simulations, this threshold has been set to 0.4 (i.e., *p_blocking_max* in Step 6 of Algorithm 1) in order to evaluate the performance of the proposed algorithm for highly loaded networks. Moreover, it is assumed that each BS is equipped with *M*_*t*_ = 2 transmit antennas, while each MS has *M*_*r*_ = 2 receive antennas. In order to simplify resource allocation, it has been assumed that in all simulation scenarios *U*_*n*_ = *U*. This practically means that equal number of subcarriers is allocated per MS.

**Figure 4:**
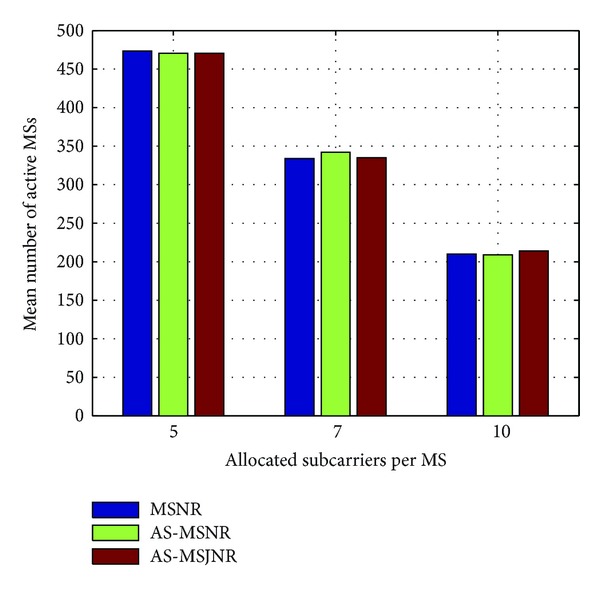
Mean number of active MSs for QPSK modulation and one tier of cells around the central cell.

**Figure 5:**
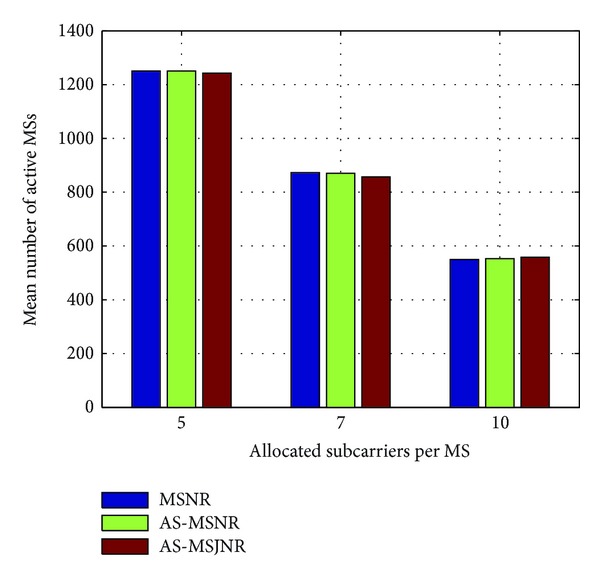
Mean number of active MSs for QPSK modulation and two tiers of cells around the central cell.

**Figure 6:**
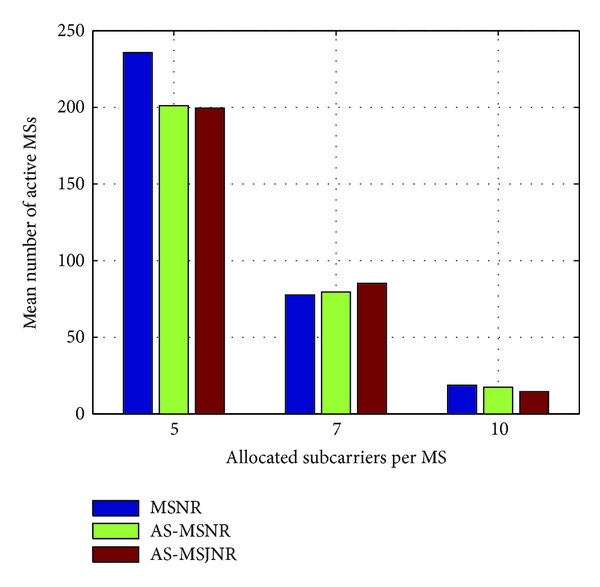
Mean number of active MSs for 16-QAM modulation and one tier of cells around the central cell.

**Figure 7:**
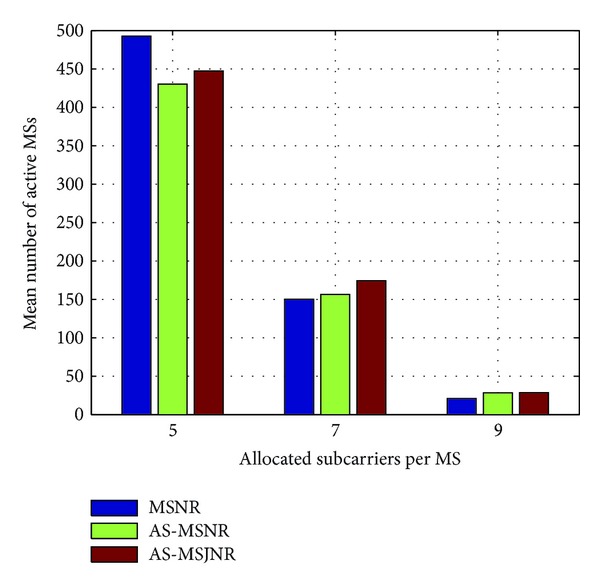
Mean number of active MSs for 16-QAM modulation and two tiers of cells around the central cell.

In Figures 4, 5, 6, and 7, the number of allocated subcarriers per MS varies from 5 to 10 (horizontal axis), while the mean number of active MSs in the network topology is presented in the vertical axis. Therefore, five types of services can be supported: 781.25/1093.75/1562.5 Kbps (QPSK modulation) and 1562.5/2187.5/3125 Kbps (16-QAM modulation). The SINR value per subcarrier for acceptable QoS has been set to 9.6/16.4 dB for QPSK/16-QAM modulation, respectively [15]. In each group of bars the first legend (i.e., MSNR) denotes subcarrier allocation based on the Maximization of the desired MS's Signal to Noise Ratio (MSNR). In this case, with respect to Algorithm 1, only steps 1, 2, 5, and 6 are performed. Moreover, the available set of subcarriers will be given by *A*_SC_ ← **C**_sec,*k*_ in line 3. The second legend in Figures 4, 5, 6, and 7 (i.e., AS-MSNR) denotes subcarrier allocation with adjacent sectorization based on MSNR while the third label (AS-MSJNR) denotes the proposed subcarrier allocation strategy. The term adjacent sectorization implies that at the initial stage of the algorithm (i.e., Step 2) subcarrier allocation is based on the joint set of the available subcarriers of the considered sector and its adjacent sectors as well, as previously explained.

If, however, the elements of this set are fewer compared to *U*_*n*_, then subcarrier allocation is based either on MSNR (i.e., AS-MSNR) or on the proposed Maximization of SJNR technique (i.e., AS-MSJNR). Note that in both cases all steps of Algorithm 1 are performed, and the only difference between AS-MSNR and AS-MSJNR strategies is subcarrier allocation of Step 4 (line 19). In the first case (i.e., AS-MSNR), then the selected subcarrier is the one that maximizes the Frobenius norm of the corresponding channel matrix, as in line 6 of Step 2. In the second case (i.e., AS-MSJNR), the selected subcarrier is the one that maximizes the SJNR ratio.

**Figure 8:**
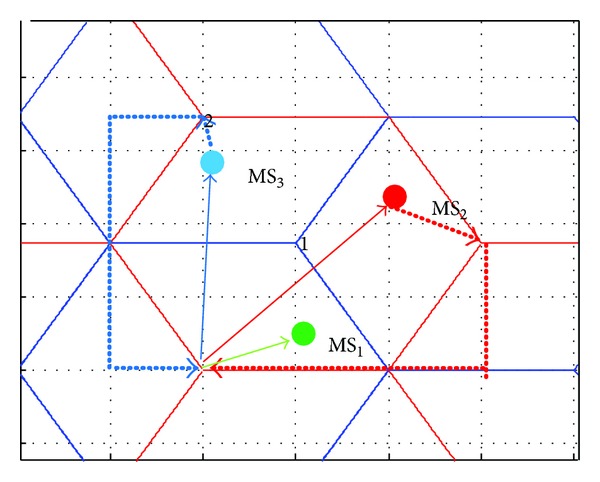
Cooperation among adjacent sectors.

As it can be observed from Figures 4 and 5, the AS-MSJNR strategy provides practically no gain compared to the other two approaches. For QPSK modulation, the required *E*_*b*_/*N*_*o*_ value is significantly lower compared to the corresponding value for 16-QAM modulation; hence, intercell interference is reduced. For 1 tier of cells around the central cell (i.e., Figure 4) and ten allocated subcarriers per MS we have 210/209/214 active MSs for the MSNR/AS-MSNR/AS-MSJNR strategies, respectively. Therefore, the AS-MSJNR strategy provides an almost 2% gain compared to the other two approaches. However, from Figures 7 and 8, it can be seen that for 16-QAM modulation the AS-MSJNR strategy provides increased number of active MSs in the network compared to the other two techniques. In particular, for 1 tier of cells (i.e., Figure 6) and 7 allocated subcarriers per MS, there are 77.6/79.5/85.2 active MSs in the network for the MSNR/AS-MSNR/AS-MSJNR strategies. Therefore, we have a 7%/9.8% gain of the AS-MSJNR strategy compared to the AS-MSNR/MSNR strategies, respectively. This gain is further increased when considering two tiers of cells. In this case, the corresponding mean number of active MSs is 150.2/155.6/174.5 for the three techniques, respectively; therefore now the corresponding gain values are increased to 12%/16%.

Note, however, that for five and ten allocated subcarriers per MS the gain of the AS-MSJNR strategy is practically negligible in both cases. In the first case, this is due to the reduced downlink transmission power per MS, since there are only five allocated subcarriers. In the second case, although there is increased downlink transmission power per MS as there are ten allocated subcarriers, the total number of MSs is reduced. Hence, as a consequence, intercell interference is also reduced and MSJNR strategy cannot be beneficial in this case.

Finally, it is interesting to note that for 16-QAM modulation and five allocated subcarriers per MS, then as it can be observed from Figures 6 and 7, the MSNR strategy outperforms the AS-MSNR and AS-MSJNR strategies. In this case, as it was explained in Section 3, for high number of accepted MSs the first *S* allocated subcarriers per set of adjacent sectors will be equally distributed among these sectors (i.e., *S*/3 subcarriers per sector). Therefore, for these first *S* subcarriers reuse factor is reduced to 1/3. As a consequence, intercell interference is increased, thus deteriorating the performance of the AS-MSNR and AS-MSJNR strategies compared to MSNR strategy, where in the latter case all allocated subcarriers are randomly distributed per hexagonal cell.

### 4.2. Practical Considerations

At this point, some practical considerations should be noted when employing the proposed subcarrier allocation algorithm in realistic next generation wireless networks. As derived by (9), an MS should be in position to estimate not only the channel matrix from its serving sector, but also the channel matrices from the serving sectors of all other interfering MSs as well. Then, assuming cooperation among different BSs or among clusters of BSs through Radio Network Controllers (RNCs) this information is exchanged in the network in order to formulate the SJNR ratios for all MSs and subcarriers. Therefore, it becomes apparent that the proposed allocation strategy would impose a significant computational and signaling burden in the network.

For this reason, an alternate approach is proposed, which simplifies the calculation of the SJNR ratios. In particular, it is assumed that an active MS should be in position to estimate the channel matrices only from potential interfering MSs that are located either in sectors that belong to the same BS as the desired MS or in adjacent sectors of the specific MS's sector. For all other BSs and sectors, the calculation of jamming values with respect to (9) is performed by considering random values for the corresponding channel matrices. Therefore, the desired MS should be in position to estimate and send back to its serving sector the channel matrices for up to four interfering MSs. Then, assuming cooperation only among adjacent BSs (i.e., up to 7 BSs), this information becomes available among each set of adjacent sectors. This procedure is illustrated in Figure 8, where MSs labeled as 2 and 3 interfere with the MS labeled as 1. The signal to the desired MS (i.e., green arrow) also propagates to the other two MSs (i.e., red and blue arrows). These individual signals are estimated and send back to MS_1_'s serving sector through the other two BSs.

**Figure 9:**
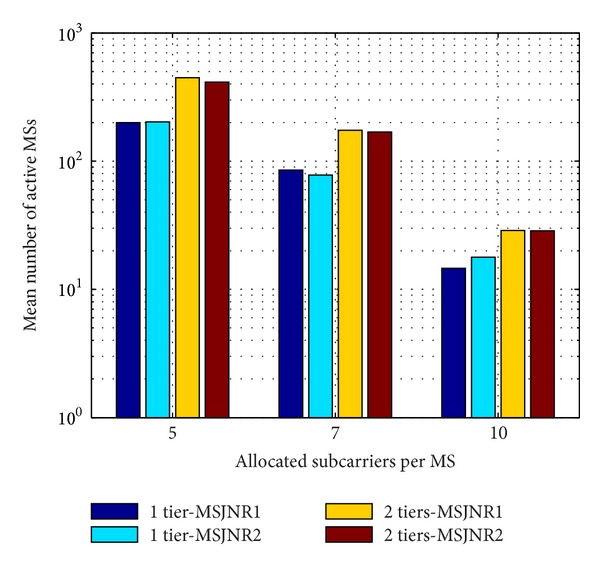
Mean number of MSs for the MSJNR1 and MSJNR2 techniques.

In Figure 9, the mean number of active MSs is presented on a logarithmic scale in the vertical axis versus the number of allocated subcarriers per MS for one tier and two tiers of cells around the central cell, considering 16-QAM modulation. The MSJNR strategy as described in Algorithm 1 where all interfering MSs are included in the denominator of (9) is denoted by MSJNR1, while the proposed variation of this technique is denoted by MSJNR2.

As it can be observed from Figure 9, these two techniques have similar performance especially in the case of two tiers of cells around the central cell; hence, it is shown that the proposed subcarrier allocation strategy can be as beneficial as its original version in (9) with significantly reduced signaling burden.

## 5. Conclusions

A new subcarrier allocation strategy for MIMO-OFDMA multicellular networks has been presented, which improves network performance in terms of mean number of active users, compared to the case of allocating subcarriers based only on the maximization of the desired MS's signal. As it was shown by the presented results, this improvement favors high data rate MSs (i.e., 16-QAM modulation and seven allocated subcarriers per MS), due to the increased levels of intercell interference in the network. Moreover, a variation of the proposed technique was presented as well, which significantly reduces signaling burden and overall complexity in cases of multicellular orientations. In this case, channel estimation and feedback to the BS are limited for up to four interfering MSs, as explained in the second part of Section 4.

Ongoing work includes among others the extension of the presented results for other MIMO-OFDMA orientations (i.e., more tiers of cells, different MIMO configurations, etc.) with limited feedback, as well as the evaluation of the proposed strategy in relay networks.

## Figures and Tables

**Algorithm 1:**
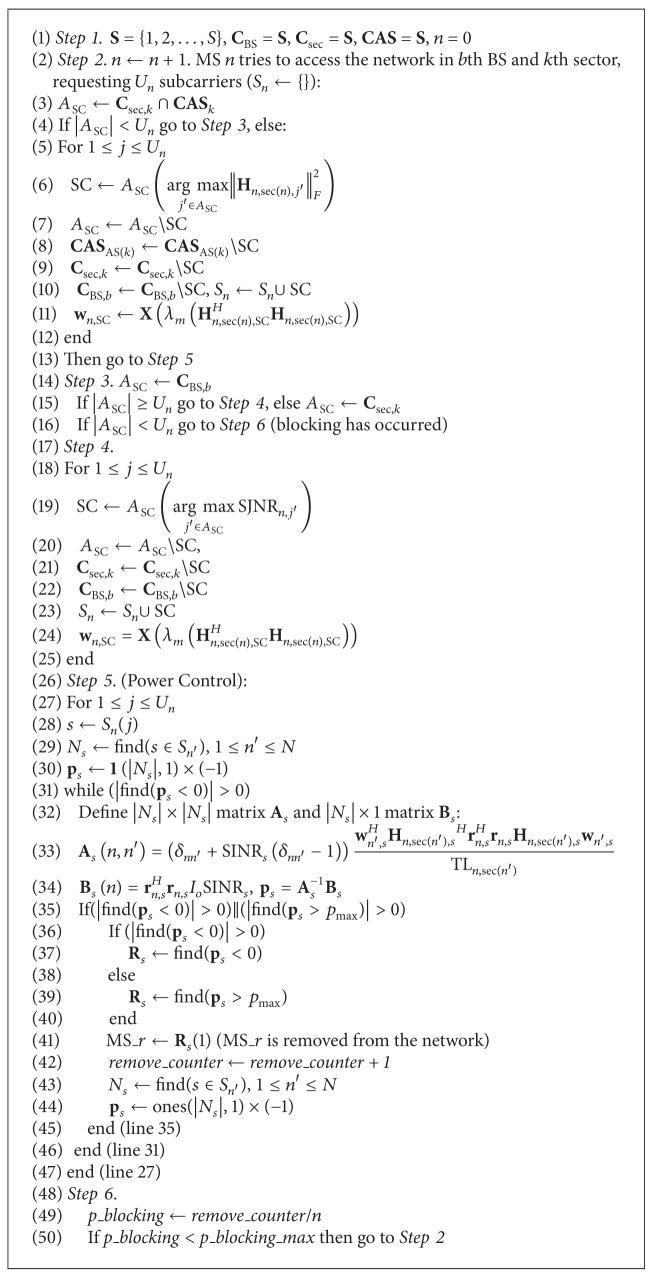
The proposed subcarrier allocation strategy.

**Table 1:** Simulation parameters.

Parameter	Value/assumption
Cell radius	1000 m
Tiers around the central cell	1, 2
Number of cells	7/19 (1/2 tiers of cells, resp.)
Carrier frequency	2.5 GHz
Modulation types	QPSK/16-QAM
BS/MS height	30/1.5 m
Propagation	Okumura-Hata, path loss exponent 3.5
Standard deviation for shadow fading	8 dB
Azimuth dispersion	Laplacian distribution, azimuth spread 5°
Maximum power per BS/MS	43/30 dBm
Radiation pattern of the antenna element	Broadside gain = 14 dBi 3 dB beamwidth = 70° Front-to-back ratio = 20 dB
Total bandwidth	10 MHz
Thermal noise level at MSs (*I*_*o*_)	−104 dBm
Total subcarriers per sector	128
Subcarriers per MS	5, 7, 10
Bandwidth per subcarrier	78.125 KHz
